# Performance evaluation of ZnSnN_2_ solar cells with Si back surface field using SCAPS-1D: A theoretical study

**DOI:** 10.1016/j.heliyon.2023.e20601

**Published:** 2023-10-02

**Authors:** Abdelmoumene Laidouci, Mamta, V.N. Singh, Pratap Kumar Dakua, Deepak Kumar Panda

**Affiliations:** a Faculty of Sciences, University of Blida 1, 09000, Blida, Algeria; b Academy of Scientific and Innovative Research (AcSIR), Ghaziabad, Uttar Pradesh, 201002, India; c Indian Reference Materials (BND) Division, CSIR-National Physical Laboratory, Dr. K. S. Krishnan Marg, New Delhi, 110012, India; d Department of ECE, Vignan's Institute of Information Technology (A), Duvada, Vishakapatnam, AP, 530049, India; e School of Electronics Engineering VIT-AP University, 522237, India

**Keywords:** ZnSnN_2_, Silicon, SCAPS 1D, Thin films, Thickness, BSF

## Abstract

The earth-abundant semiconductor zinc tin nitride (ZnSnN_2_) has garnered significant attention as a prospective material in photovoltaic and lighting applications, primarily due to its tunable narrow bandgap and high absorption coefficient. This study focuses on a numerical investigation of ZnSnN_2_ solar cell structures using the SCAPS 1-D software. The objective is to analyze the influence of various physical and geometrical parameters on solar cell performance. These parameters include the thicknesses of the ZnO window layer, CdS buffer layer, ZnSnN_2_ absorber layer, and Si back surface field layer (BSF), as well as operating temperature, series and shunt resistances (R_S_ and R_sh_), absorber layer defect density, interface defects, and the generation-recombination profile of the n-ZnO:Al/n-CdS/p-ZnSnN_2_/p-Si/Mo structure. We have evaluated the capabilities of this novel material absorber by investigating its performance across a range of thicknesses. We have started with ultrathin absorber thicknesses and gradually increased them to thicker levels to determine the optimal thickness for achieving high efficiency. Under optimal conditions, a thin solar cell with a thickness (w_p_) of 1 μm achieved an efficiency (η) of 23.9%. In a practical solar cell operating at room temperature, optimal parameters were observed with a thicker absorber layer (w_p_ = 8 μm) and a BSF width of 0.3 μm. The cell exhibited resistances of R_sh_ = 10^6^ Ω cm^2^ and R_s_ = 1 Ω cm^2^, along with a low defect density (N_t_ = 10^10^ cm^−3^) in the ZnSnN_2_ semiconductor. These factors combined to yield an impressive efficiency of 29.5%. Numerous studies on emerging ternary nitride semiconductors (Zn-IV-N_2_) have highlighted ZnSnN_2_ as a promising material for thin-film photovoltaics. This compound is appealing due to its abundance, non-toxicity, and cost-effectiveness. Unlike conventional solar cells that depend on rare, toxic, and costly elements, these components are still essential for today's solar cell technology.

## 1 Introduction

**Fig. 1:**
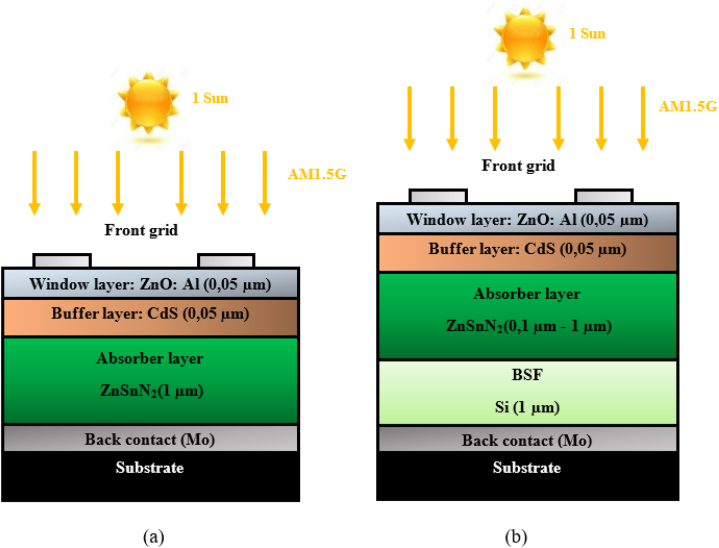
(a) Schematic structure of the thin ZnSnN_2_ solar cell without BSF, (b) Schematic structure of the thin ZnSnN_2_ SCs with BSF [27].

In recent decades, researchers have highlighted the importance of numerical and computing research through advanced programs that model and simulate devices, enabling the exploration of material properties. It yields financial benefits and stimulates investment in scientific research, particularly for predicting manufacturing and experimental outcomes [1]. Among the ternary Zn-IV-N_2_ semiconductors, ZnSnN_2_ consists of non-toxic, earth-abundant, and cost-effective elements [2]. Regarding symmetry, Zn-IV-N_2_ semiconductors resemble the wurtzite structure of III-N semiconductors [[3], [4], [5]]. They exhibit comparable electronic, optical, and polarization properties, including direct band gaps, high optical absorption coefficients, and spontaneous polarization [[6], [7], [8]]. Recent studies have highlighted In_x_Ga_1-x_N and ZnSnN_2_ as promising photovoltaic absorbers within the nitride compounds of III-N and II-IV-N_2_ materials. However, the growth of crystalline III-V materials requires expensive and complex epitaxial growth techniques. For example, using indium, gallium, and arsenic in many solar cells is undesirable due to certain elements' scarcity, cost, and toxicity [[9], [10], [11], [12], [13]]. *p*-SnO/n-ZnSnN_2_ and p-Si/n-ZnSnN_2_ junctions were recently successfully fabricated for photovoltaic applications [14,15]. A buffer layer of Al_2_O_3_ was employed in the *p*-SnO/n-ZnSnN_2_ solar cell to enhance its efficiency, which was boosted to 1.54% [15]. Furthermore, a PV device based on ZnSnN_2_ was also theoretically analyzed, exhibiting a ∼23% efficiency without defects [[16], [17], [18]]. Several forms of ZnSnN_2_ films have been synthesized, including polycrystalline and monocrystalline films, on monocrystalline substrates such as (sapphire “Al_2_O_3_”, GaN, Yttria-stabilized Zircona “YSZ”, etc.) by radio frequency (RF) sputter deposition [19], Molecular beam epitaxy (MBE) [20], and plasma-assisted vapor–liquid–solid technique (VLS) [21]. According to experimental findings, it has been determined that ZnSnN_2_ possesses a direct bandgap of approximately 1.7 eV [22]. Moreover, theoretical investigations show that the direct bandgap of ZnSnN_2_ can vary from 1 to 2 eV depending on the degree of disorder in which it crystallizes [11,23]. Experimental results have demonstrated that ZnSnN_2_ behaves as an n-type degenerate semiconductor with a high concentration of electrons but low mobility [24]. While the issue of p-type doping has been a challenge, we have considered it and utilized ZnSnN_2_ as a p-type semiconductor [4,25,26]. This study investigated various physical and geometrical parameters affecting solar cells, such as the thicknesses of the ZnO window layer, CdS buffer layer, ZnSnN_2_ absorber layer, and Si back surface field layer (BSF), as well as operating temperature, series and shunt resistances, absorber layer defect density, interface defects, and the generation-recombination profile and their effect on the electrical parameters. Fig. 1(b) shows the proposed design of the thin ZnSnN_2_ solar cells. The ZnSnN_2_ films used in this study have orthorhombic lattice parameters of a = 0.585 nm, b = 0.676 nm, and c = 0.558 nm [21]. To minimize strain, CdS and Si were selected with a low strain of 0.37% and 7.17%, respectively, for ZnSnN_2_.This new structure has been proposed by H. Heriche et al. (n-ZnO:Al/n-CdS/p-CIGS/p-Si/Mo) [27]. Additionally, silicon has been utilized as a secondary absorber and a Back Surface Field (BSF) to improve efficiency. On the other hand, an ohmic back contact was created by depositing molybdenum (Mo) on a coated glass substrate.

## 2 Materials and methods

**Fig. 2:**
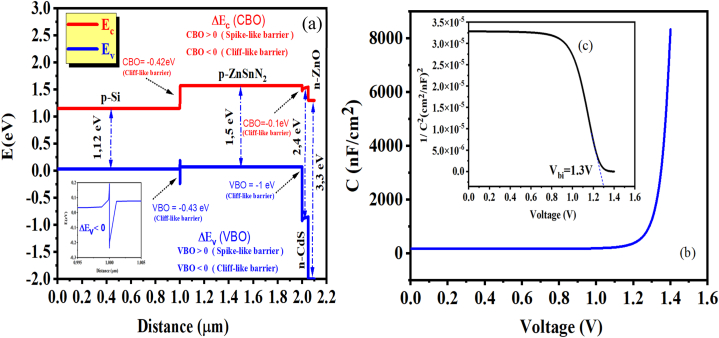
(a) Energy band diagram of the new thin ZnSnN_2_ solar cells, (b) C–V characteristics, and (c) Mott-Schottky plot.

The energy band diagram for the new thin ZnSnN_2_ SCs has been obtained using SCAPS-1D software, as depicted in Fig. 2 (a). Fig. 1(b) shows the proposed design of the thin ZnSnN_2_ photovoltaic cell. This section outlines the physical models utilized in the study, where SCAPS-1D employs a numerical method called Finite Difference Method (FDM) to solve semiconductor equations, Poisson's equation, and continuity equations, which are represented by equations (1), (2), (3) [28]. For carriers, the Poisson equation is as follows:

(1) $$\frac{\partial^{2}}{\partial x^{2}}\psi \left(x\right)=\frac{e}{\varepsilon_{0}\varepsilon_{r}}\left(p\left(x\right)-n\left(x\right)+N_{d}-N_{a}+\rho_{p}-\rho_{n}\right)$$

where Ψ is the electrostatic potential, n is the electron concentration, p is the hole concentration, ε_r_ is the relative permittivity, e is the electrical charge, N_d_ is the donor concentration, ε_0_ is the vacuum permittivity, N_a_ is the acceptor concentration, ρ_n,_ and ρ_p_ are electrons and holes distribution. The continuity equations for carriers are:

(2) $$\frac{1}{q}\;\frac{dJ_{p}}{\text{dx}}=G\left(x\right)-R\left(x\right)$$

(3) $$\frac{1}{q}\;\frac{dJ_{n}}{\text{dx}}=-G\left(x\right)+R\left(x\right)$$

where G(x) is the charge generation rate, and R(x) is the charge recombination rate.

According to transport theory, the drift-diffusion current can be mathematically expressed by the following equations (4), (5):

(4) $$J_{n}=D_{n}\frac{\text{dn}}{\text{dx}}+\mu_{n}n\frac{d\psi }{\text{dx}}$$

(5) $$J_{p}=D_{p}\frac{\text{dp}}{\text{dx}}+\mu_{p}p\frac{d\psi }{\text{dx}}$$

where μ_n_ and μ_p_ are electron and hole mobilities, J_n_ and J_p_ are electron and hole current densities, D_n_ and D_p_ are electron and hole diffusion coefficients, respectively.

The EQE (External Quantum Efficiency) is determined by integrating the following expression (6) [29]:

(6) $$J_{\text{ph}}\;\left(\lambda \right)=q\;\int_{\lambda_{1}}^{\lambda_{2}}F\left(\lambda \right)\;\text{EQE}\left(\lambda \right)\;d\lambda$$

where J_ph_ (λ) is the total photocurrent density, and F(λ) is the photon flux of the incident spectrum.

In the single-diode model, the current is typically expressed by the following equation (7) [30]:

(7) $$I=I_{L}-I_{0}\left(\exp^{\frac{q\left(V+IR_{s}\right)}{\text{nKT}}}-1\right)-\frac{V+IR_{s}}{R_{\text{sh}}}$$

where I_L_ is the load current, R_s_ is the series resistance, R_sh_ is the shunt resistance, I_0_ is the reverse saturation current, and I is the output current. It is also possible to represent I_0_ as follows in equation (8) [30,31]:

(8) $$I_{0}=q\;A\;\left(\frac{D_{n}}{L_{n}}\;\frac{n_{i}^{2}}{N_{A}}+\frac{D_{p}}{L_{p}}\;\frac{n_{i}^{2}}{N_{D}}\right)$$

where A is the p-n junction area, L_n_ and L_p_ are electron and hole diffusion lengths, and n_i_ intrinsic carrier concentration, respectively.

The V_oc_ is mathematically represented by the following equation (9) [30]:

(9) $$V_{\text{oc}}=\frac{\text{KT}}{q}\ln\left(\frac{I_{L}}{I_{0}}+1\right)$$

where T is temperature, and K is Boltzmann's constant, respectively.

The empirical fill factor can be accurately expressed by equation (10) as follows [31,32]:

(10) $$\text{FF}=\frac{v_{\text{oc}}-\ln\left(v_{\text{oc}}+0.72\right)}{v_{\text{oc}}+1}$$

where $v_{oc}$ is normalized V_oc_ ($v_{oc}=$ V_oc_/V_t_), and V_t_ is the thermal voltage (V_t_ = kT/q).

Varshni's law is an empirical equation that quantifies the relationship between temperature and bandgap energy in semiconductors. It is given by equation (11) as follows [17]:

(11) $$\text{Eg}\;\left(T\right)=\text{Eg}\;\left(0K\right)-\frac{\alpha \;T^{2}}{T+\beta }$$

where α, β, and Eg (0 K) are material constants.

Recent studies have illustrated that the strategic introduction of lithium or barium into ZnSnN_2_ can effectively transform it into a p-type semiconductor, as confirmed by experimental evidence [26,33]. Our study quantitatively assessed relevant parameters, including carrier density, dislocation density, and mobility. This was achieved through Hall effect measurements conducted on ZnSn_x_Ge_1-x_N_2_ films with a specific alloy composition (x = 1) and X-ray diffraction (XRD) analysis on Li: ZTN thin films, as detailed in references [33,34]. The strain was calculated using the relation from Ref. [35], where a_e_ is the lattice parameter of the epitaxial layer and a_s_ is the lattice parameter of the substrate. We present an overview of our simulation input parameters in Table 1, Table 2, Table 3 [[16], [17], [18],^26^,^27^,^33^,^34^,[36], [37], [38], [39], [40], [41], [42]]. equations (12), (13) describing the band offset between the conduction and valence bands are as follows [43,44]:

(12) $$\Delta \;E_{C}\left(\text{CBO}\right)=\chi^{{\text{ZnSnN}}_{2}}-\chi^{\text{CdS}}\;\left({\text{ZnSnN}}_{2}-\text{buffer}\right)$$

**Table 1:** Input parameters used in the study and their values.

Parameters	Materials proprities			
	ZnSnN_2_ (p)	CdS (n)	ZnO: Al (n)	Si (p)
ε	15	10	9	11.9
E_g_ (eV)	1.5	2.4	3.3	1.12
E_a_ (eV)	4.1	4.2	4.45	4.05
N_c_ (cm^−3^)	1.2 × 10^18^	2.2 × 10^18^	2.2 × 10^18^	2.80 × 10^19^
N_v_ (cm^−3^)	7.8 × 10^19^	1.8 × 10^19^	1.8 × 10^19^	2.65 × 10^19^
μ_e_ (cm^2^V–^1^S^−1^)	12.68	100	100	1450
μ_h_ (cm^2^V–^1^S^−1^)	5.26	25	25	500
N_d_ (cm^−3^)	0	1 × 10^17^	1 × 10^20^	0
N_a_ (cm^−3^)	9.47 × 10^19^	0	0	1.00 × 10^20^
Thickness d (μm)	Variable	Variable	Variable	Variable
Lattice parameter a (nm)	0.585	0.582	/	0.543
Defect density N_t_ (cm^−3^)	Variable	10^16^(A)	10^16^(A)	10^14^(D)
α (cm^−1^)	from [17]	from [36]	from [36]	from [27]
$\varepsilon_{\text{xx}}=\frac{a_{s}-a_{e}}{a_{e}}$	0.515%		/	7.17% (ZnSnN_2_)

**Table 2:** Defect parameters used at CdS/ZnSnN_2_ and ZnSnN_2_/Si interfaces.

Parameters	CdS/ZnSnN_2_ interface	ZnSnN_2_/Si interface
Defect type	Neutral	Neutral
Capture cross-section of electrons (cm^2^)	10^–19^	10^–19^
Capture cross-section of holes (cm^2^)	10^–19^	10^–19^
Reference for defect energy level E_t_	above the highest E_v_	above the highest E_v_
Energy with respect to reference (eV)	0.6	0.6
Total density (cm^−2^)	Variable	Variable

**Table 3:** Simulation parameters for front and back contacts.

Contact properties	Front	Back
Metal Work Function	Flat Band	4.95
Surface recombination velocity of electron ‘S_n_’ (cm/s)	10^5^	10^7^
Surface recombination velocity of hole ‘S_p_’ (cm/s)	10^7^	10^5^
Reflectivity ‘R’ (%)	10	90
Working temperature (K)	280–400	

Hence, the valence band alignment is as follows:

(13) $$\Delta \;E_{V}\left(\text{VBO}\right)=\left(\chi^{{\text{ZnSnN}}_{2}}+{E_{g}}^{{\text{ZnSnN}}_{2}}\right)-\left(\chi^{\text{CdS}}+{E_{g}}^{\text{CdS}}\right)$$

where E_g_ is the bandgap energy and χ is the electron affinity.

The built-in potential (V_bi_) of an n-p junction device, considering variations in doping density, is determined by the following equation (14) [45]:

(14) $$V_{\text{bi}}=\frac{\text{KT}}{q}\ln\left(\frac{N_{A}N_{D}}{n_{i}^{2}}\right)$$

In solar cells, the V_oc_ is typically lower than V_bi_ because it is affected by factors such as recombination losses and series resistance [45]. The value of the built-in potential in an n-p junction can also be obtained from the intercept of the Mott-Schottky plot, which is determined using the following equation (15) [45]:

(15) $$\frac{1}{C^{2}}=\frac{2}{q\varepsilon_{r}\varepsilon_{0}\;N}\;\left(\;V-V_{\text{bi}}-V_{t}\right)$$

where C represents the space charge capacitance per unit area, and N represents the carrier concentration.

## 3 Results and discussion

Throughout this study, in all simulations, we used ambient temperature, one sun AM1.5G, and considered the flat-band conditions on the front contact. Fig. 1 (b) depicts the thin ZnSnN_2_/Si solar cell's structure proposed by H. Heriche et al. [27]. This innovative design replaces the conventional copper indium gallium diselenide (CIGS) material with ZnSnN_2_, an earth-abundant semiconductor. This substitution's main objective is to avoid using toxic and expensive elements, namely indium, selenium, and gallium. This substitution addresses the environmental concerns and cost implications associated with these materials.

Moreover, the objective of this study extends beyond the implementation of ultrathin solar cells; it also delves into the exploration of the potential benefits that a thick absorber layer can offer in achieving high efficiency. As depicted in Fig. 2 (a), the magnitude of the band offset in a buffer-absorber and absorber-BSF layers system is evident. It is crucial to note that the difference in electron affinity between the respective layers intricately determines this magnitude. The band offset refers to the energy difference between adjacent materials' valence and conduction bands in a heterojunction. Moreover, the statement explains that the variation in electron affinity between the layers leads to the emergence of distinct structural characteristics within the layer system. Specifically, positive band offsets give rise to spike-like formations, while negative band offsets result in cliff-like configurations. These descriptions effectively visually represent the band offset's impact on the layer system's structure [43,44]. Developing a cliff-like band structure is a remarkable feature of the ZnSnN_2_/CdS interface. The conduction band offset (CBO) of −0.1 eV suggests that the conduction band energy levels of ZnSnN_2_ are slightly lower than those of CdS at the interface. This CBO facilitates electron transfer from CdS to ZnSnN_2_, crucial for efficient charge separation in various optoelectronic devices.

Additionally, the valence band offset (VBO) of −1 eV indicates an upward shift in energy levels for the valence band of ZnSnN_2_ compared to CdS. This VBO promotes hole transfer from ZnSnN_2_ to CdS, enhancing charge carrier separation. When both band offsets are negative, it increases carrier recombination, which in turn causes a decrease in V_oc_ [44]. These observations provide valuable insights into the band alignment and electronic structure of the ZnSnN_2_/CdS interface, which can be exploited to design and optimize novel semiconductor heterostructures. At the Si/ZnSnN_2_ interface, an intriguing cliff-like band structure is observed. The CBO of −0.42 eV indicates a high downward shift in the energy levels of Si compared to ZnSnN_2_. This CBO facilitates the transfer of electrons from ZnSnN_2_ to Si, enabling efficient electron injection or collection in devices such as photovoltaic cells.

On the other hand, positive band offsets may restrict electron transport, leading to increased electron trapping due to potential barriers in the device, which lowers the cell's J_sc_ and PCE (%) [44]. The valence band offset (VBO) of −0.43 eV reveals an upward shift in the valence band energy levels of ZnSnN_2_ relative to Si. This VBO promotes hole transfer from Si to ZnSnN_2_, facilitating the separation and transport of positive charge carriers. The C–V characteristics, Mott-Schottky plot, and the obtained results are depicted in Fig. 2 (b,c). Based on our comprehensive analysis, the Mott-Schottky plot reveals an extracted built-in potential value of approximately 1.3 V. The frequency used in the simulation is 1 MHz. As illustrated in Fig. 2 (b,c), the capacitance value remained constant when the voltage ranged from 0 to 1 V. However, the capacitance underwent a significant and rapid change beyond this threshold.

### 3.1 Impact of window layer and buffer layer thicknesses on photovoltaic performance

**Fig. 3:**
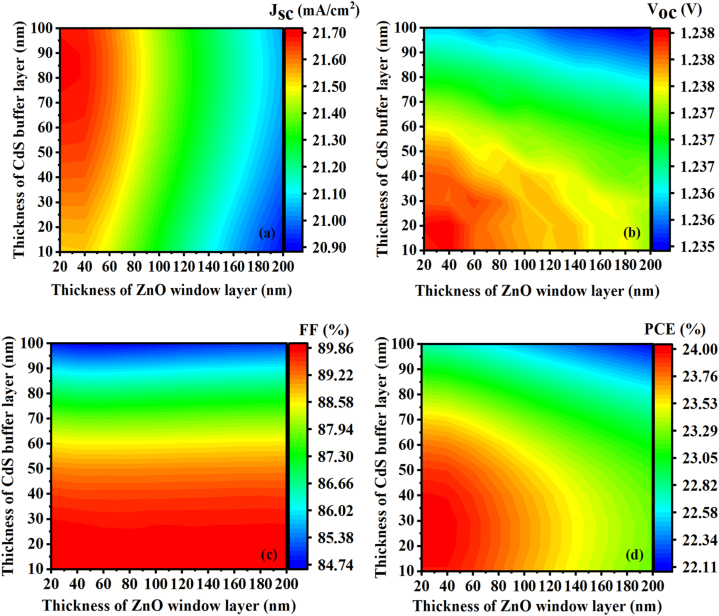
Contour plot of the effect of the thickness of the ZnO window layer and the thickness of the CdS buffer layer on (a) Jsc, (b) Voc, (c) FF, and (d) η.

The window and buffer layers play a significant role in solar cell performance. To improve performance, ZnO window layer and CdS buffer layer thicknesses have been varied from 20 to 200 nm and 10–100 nm, respectively. Fig. 3 illustrates the impact of window layer and buffer layer thicknesses on the electrical parameters of the ZnSnN_2_-based solar cell at ambient temperature. It can be observed that the variation in the thicknesses of the ZnO window layer and CdS buffer layer affects the J_SC_ (Fig. 3(a)), V_OC_ (Fig. 3(b)), FF (Fig. 3(c)), and PCE (Fig. 3(d)) of the solar cell. In the case of the J_SC_ (Fig. 3(a)), the results showed a maximum value of over 21.70 mA/cm^2^ when the window thickness was ≤60 nm and the buffer thickness was ≥50 nm. For the V_oc_ (Fig. 3(b)), the results indicated a maximum value of over 1.238 V when the window thickness was ≤80 nm and the buffer thickness was ≥60 nm. Regarding the FF (Fig. 3(c)), the results demonstrated a maximum value of over 89.86% when the buffer thickness was ≤60 nm, regardless of the window thickness. Our findings suggest that a maximum efficiency of 23.9% can be achieved with a ZnO window layer thickness of approximately 50 nm and a CdS buffer layer thickness of around 50 nm. It is important to note that an excessively thin layer can lead to leakage current, while an overly thick layer may result in a low carrier separation rate [46]. Thus, window and buffer layers can impact photovoltaic cell efficiency, stability, and durability, and optimizing them can lead to improved photovoltaic performance.

### 3.2 Influence of absorber layer thickness on photovoltaic characteristics

**Fig. 4:**
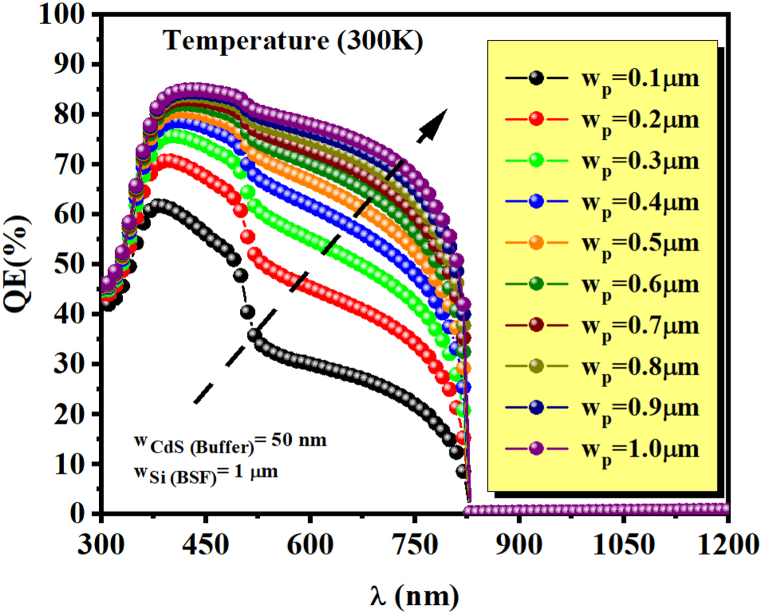
Quantum efficiency at 27 °C (300 K) as a function of ZnSnN_2_ absorber layer thickness.

**Fig. 5:**
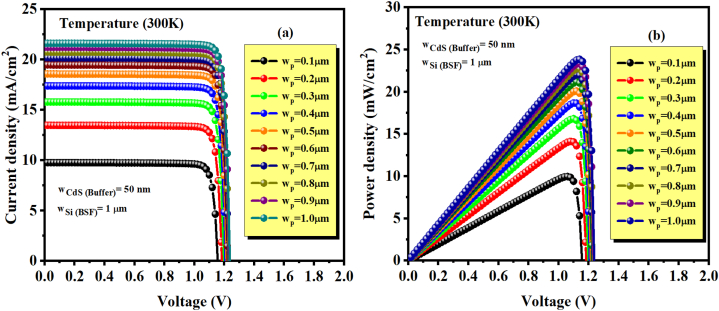
(a) J-V curves for different w_p_ (w_ZnSnN2_), (b) P–V curves for different w_p_ (w_ZnSnN2_), where w_n_ (w_CdS_) = 50 nm at 300 K.

Fig. 4 displays the ultrathin solar cell structure's quantum efficiency (QE) (ZnSnN_2_/Si) with varying ZnSnN_2_ layer thicknesses ranging from 100 nm to 1 μm. The simulation results demonstrate a significant improvement in the quantum efficiency (QE) with increased absorber thickness of ZnSnN_2_. This enhancement is observed across a wavelength range of 300 nm–826 nm, where 826 nm corresponds to the energy bandgap of ZnSnN_2_. However, no light will be absorbed beyond this wavelength due to insufficient photon energy. The improved quantum efficiency (QE) observed can be attributed to the enhanced absorption of photons across a wide range of wavelengths, specifically from 300 nm to 826 nm. This heightened absorption leads to a greater rate of carrier generation, increasing QE. The phenomenon can be explained by the increasing thickness of the absorber layer, allowing for the absorption of a larger number of photons and consequently generating a greater number of electron-hole pairs. This ultimately enhances the overall quantum efficiency [42]. In addition, the insertion of silicon as the Back Surface Field layer (BSF) significantly enhances the solar cell's performance by creating an electric field on the rear face. This electric field serves to lower the surface recombination velocity (SRV), which refers to the rate at which minority carriers (electrons or holes) recombine with opposite charge carriers at the surface of the solar cell. By incorporating silicon as the BSF layer, a potential barrier impedes the migration of minority carriers toward the surface. This barrier reduces the chances of recombination occurring at the surface, thereby minimizing the loss of carriers and improving the overall electrical characteristics of the solar cell. The reduction in surface recombination improves the efficiency of the solar cell by increasing the lifetime of the minority carriers and maximizing their chances of reaching the p-n junction, where they can contribute to the generation of electric current. The silicon BSF layer enhances the solar cell's overall performance and power output by minimizing the recombination losses at the surface [47]. Fig. 5 (a and b), at ambient temperature, w_ZnO_ = 50 nm, w_CdS_ = 50 nm, and without defect in p-type ZnSnN_2_, we show the variation of current-voltage characteristics J(V) of the studied ZnSnN_2_ solar cell and the output power for different thickness w_p_ (ZnSnN_2_) of our proposed structure. A thinner absorbing layer causes the lower photocurrent, and a too-thick absorber layer increases series resistance, material consumption, and, therefore, the price per unit of generated power. According to our results, increasing absorber thickness gradually leads to a growing shape of the curves.

**Fig. 6:**
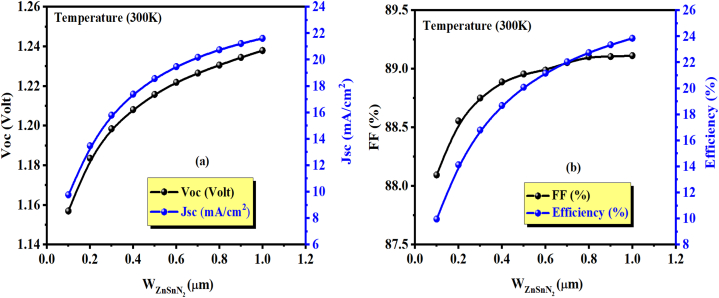
(a) Variation of V_OC_ and J_SC_, (b) Variation of FF (%) and η (%) as a function of ZnSnN_2_ absorber layer thickness.

The results show that the variation of w_p_ from 0.1 μm to 1 μm leads to increased improvement in the solar cell's performance. The enhancement of J_sc_ and the efficiency is because of the absorbed photons that contribute to the generation of carriers. According to the results shown in Table 4, Fig. 5 (a and b), and Fig. 6 (a and b), a significant improvement has been noted in the yield from 9.96% for 0.1 μm to 23.9% for 1 μm (Maximum power and efficiency are identical under AM1.5G spectrum conditions). An increment of J_sc_ from 09.77 mA/cm^2^ for 0.1 μm to 21.61 mA/cm^2^ for 1 μm and V_oc_ from 1.1569 V for 0.1 μm to 1.2379 V for 1 μm. However, the increase in FF is not significant. It is also possible that the additional thickness of the absorber layer would result in a higher V_oc_. Thus, increasing the number of charge carriers in a solar cell can cause an increase in the built-in potential across its p-n junction. The built-in potential is the potential difference across the junction without any external bias or sunlight and is influenced by the doping levels and energy bandgap of the materials used in the cell. A higher built-in potential results in a higher open circuit voltage. Hence, as the absorber thickness increases, more electron-hole pairs are generated, leading to a higher concentration of charge carriers. This results in an increase in the built-in potential and subsequently raises the open circuit voltage of the solar cell [48].

**Table 4:** Impact of absorber layer thickness on PV cell parameters of ZnSnN_2_ at 300 K.

w_p_ (μm)	Voc (V)	Jsc (mA/cm^2^)	FF (%)	η (%)
0.1	1.1569	9.772	88.09	9.96
0.2	1.1835	13.477	88.55	14.12
0.3	1.1984	15.791	88.75	16.79
0.4	1.2080	17.392	88.88	18.67
0.5	1.2157	18.568	88.95	20.08
0.6	1.2219	19.467	88.98	21.16
0.7	1.2265	20.177	89.05	22.03
0.8	1.2306	20.749	89.10	22.75
0.9	1.2344	21.221	89.10	23.34
1	1.2379	21.617	89.11	23.9

### 3.3 Impact of operating temperature on photovoltaic performance

**Fig. 7:**
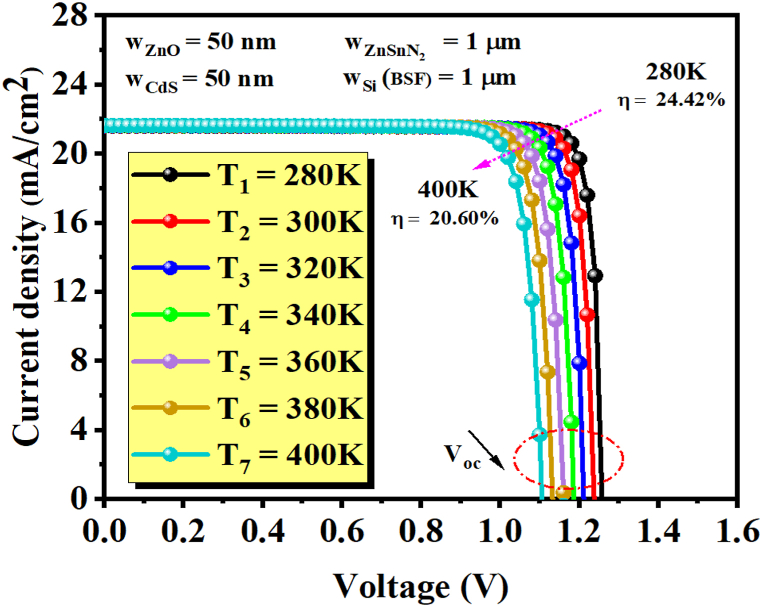
J(V) characteristics for different operating temperatures, where w_p_ (w_ZnSnN2_) = 1 μm.

**Fig. 8:**
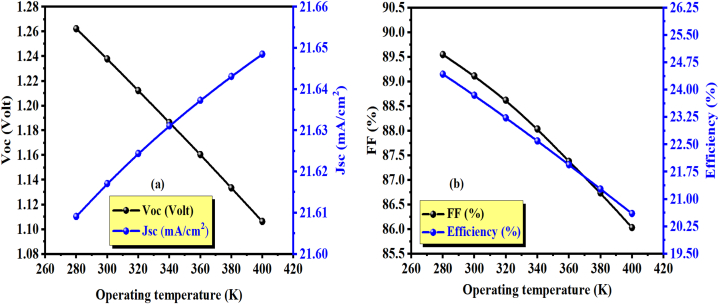
(a) Variation of V_OC_ and J_SC_, (b) Variation of FF (%) and η (%) as a function of operating temperature, where w_p_ (w_ZnSnN2_) = 1 μm.

Fig. 7 shows the current density-voltage characteristic J-V for different operating temperatures. Temperature is a key factor that affects the performance of semiconductor devices, especially solar cells. For example, as the temperature increases, the semiconductor lattice expands due to thermal expansion, changing the crystal structure. Moreover, the electron-phonon interaction becomes more significant at high temperatures. These effects reduce the band gap of the semiconductor. As a result, the charge carrier concentration increases, which influences the short-circuit current (J_sc_) [49]. In a photovoltaic cell, the reverse saturation current is highly dependent on temperature, which makes V_oc_ the most affected parameter [32,50]. According to equation (8), a diode's reverse saturation current (I_0_) strongly depends on the concentration of intrinsic charge carriers (∼n_i_^2^), which are highly affected by temperature and described by diffusion theory [51]. The output results are shown in Fig. 8 (a and b) and Table 5. A decrease in V_oc_ is observed with an increase in temperature from 1.2622 V for 280 K to 1.1063 V for 400 K. This behavior of a decrease of open-circuit voltage (V_oc_) with rising temperature is caused by an increase in the diode's reverse saturation current (I_0_), whereas the short-circuit current (J_sc_) has slightly increased. An increment in the short-circuit current (J_sc_) from 21.60 mA/cm^2^ for 280 K to 21.64 mA/cm^2^ for 400 K.

**Table 5:** Influence of operating temperature on the photovoltaic parameters.

Temperature(K)	Voc (V)	Jsc (mA/cm^2^)	FF (%)	η (%)
280	1.2622	21.609	89.548	24.42
300	1.2379	21.617	89.11	23.9
320	1.2122	21.624	88.61	23.23
340	1.1864	21.631	88.03	22.59
360	1.1605	21.637	87.37	21.94
380	1.1334	21.643	86.73	21.27
400	1.1063	21.648	86.03	20.60

Meanwhile, an increase in temperature leads to a decrease in FF. Earlier, it was stated that a drop in V_oc_ is mainly responsible for the drop in FF, while a rise in J_sc_ does not have much effect [31]. The maximum efficiency obtained is 24.42% at 280 K when w(ZnSnN_2_) = 1 μm.

### 3.4 Influence of absorber layer defect density on photovoltaic characteristics

**Fig. 9:**
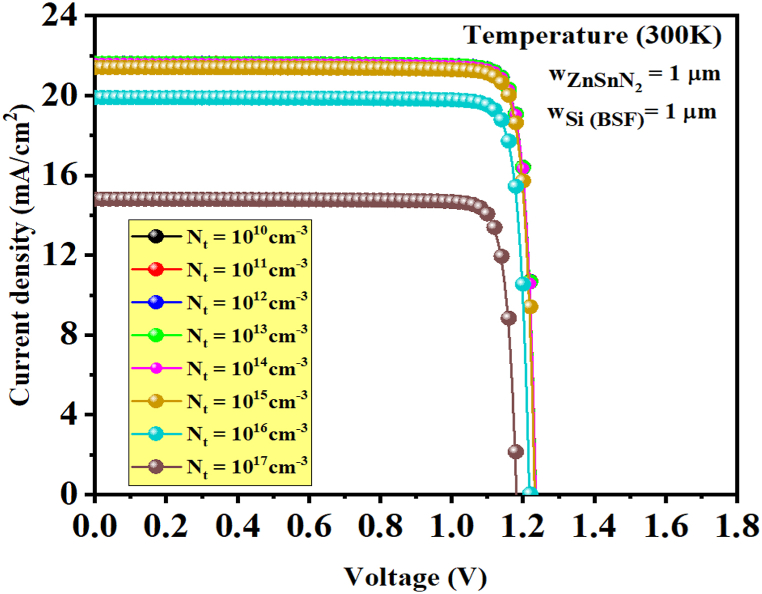
J-V curves of thin ZnSnN_2_ solar cell at the varying absorber layer defect densities (N_t_).

**Fig. 10:**
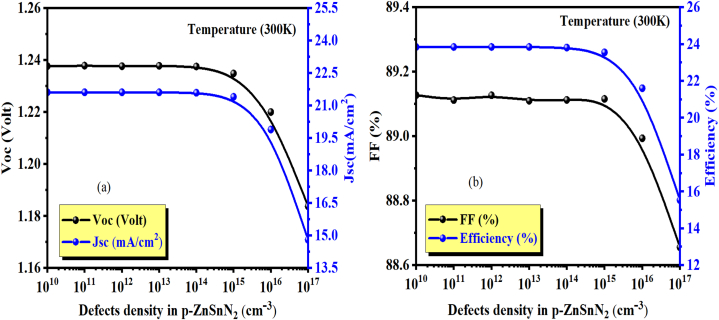
(a) Variation of V_OC_ and J_SC_, (b) Variation of FF (%) and η (%) as a function of defects density in the *p*-ZnSnN_2_ layer.

Fig. 9 shows the current density-voltage characteristic J-V for different defect states (N_t_) in the ZnSnN_2_ semiconductor. Fig. 10 (a and b) shows the extracted parameters from J-V curves (J_sc_, V_oc_, FF, and η) as a function of absorber layer defect density (N_t_). All these parameters get decreased when defects are included. In electronic devices, it is common for semiconductor defects to cause electron-hole recombination [28]. Defects in a material can create localized energy levels within the bandgap, trapping charge carriers and decreasing their lifetime. It can cause current leakage, reducing the solar cell's yield and negatively impacting its electrical characteristics [52]. The corresponding efficiencies for 10^10^ cm^−3^ and 10^17^ cm^−3^ were about 23.9% and 15.54%, respectively. These values have undergone testing to address the limited availability of experimental data for the p-type ZnSnN_2_ semiconductor. The reported total density of defects for the ZnSnN_2_ material can be found in the previous literature [4,26,33]. The improvement in the recombination process, which causes the annihilation of the charge carriers, is primarily responsible for the decrease in performance with an increase in defect density. A lower defect density results in a higher carrier diffusion length and a lower recombination rate, which improves PV performance.

### 3.5 Influence of defect density at CdS/ZnSnN_2_ and ZnSnN_2_/Si interfaces

**Fig. 11:**
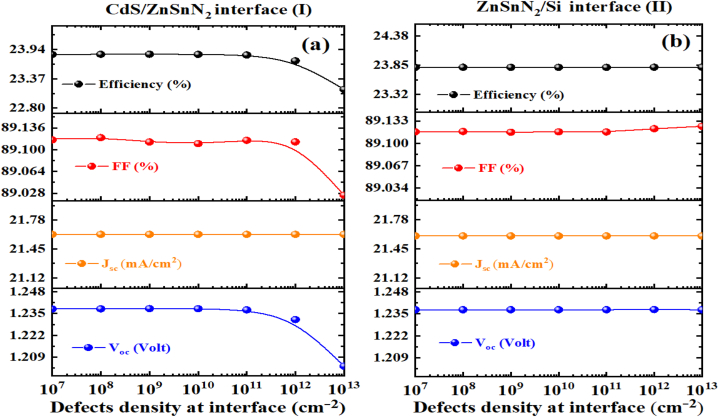
Effects of defect density at the (a) CdS/ZnSnN_2_ and (b) ZnSnN_2_/Si interfaces on photovoltaic performance.

In this section, we performed a numerical simulation to explore the influence of defect density on the interfaces of CdS/ZnSnN_2_ and ZnSnN_2_/Si in ZnSnN_2_ solar cells. Structural defects in heterojunction PV devices can result in interfacial defects. Hence, it is crucial to investigate the impact of these defects on the solar output parameters. Our findings indicate that defects at the interface I (Buffer/Absorber) can increase the likelihood of carrier trapping and R_s_ in the HJT [47]. Fig. 11(a) shows the effect of defect density at the CdS/ZnSnN_2_ interface on the PV parameters. The neutral defect density at the CdS/ZnSnN_2_ interface varies from 10^7^ to 10^13^ cm^−2^. The corresponding parameters can be found in Table .2. In Fig. 11(a), V_oc_ decreases from 1.2377 to 1.2037 V as the CdS/ZnSnN_2_ interface defect density ranges from 10^7^ to 10^13^ cm^−2^. While J_sc_ remains unchanged as the CdS/ZnSnN_2_ interface defect density ranges from 10^7^ to 10^13^ cm^−2^. The high defect density at the buffer/absorber interface increases the series resistance of the heterojunction solar cell [47]. Our results show a slight decrease in the fill factor, dropping from 89.12% to 89.02%, corresponding to a variation in the CdS/ZnSnN_2_ interface defect density from 10^7^ to 10^13^ cm^−2^. The efficiency decreased from 23.9% to 23.15% as the CdS/ZnSnN_2_ interface defect density increased from 10^7^ to 10^13^ cm^−2^. This increase in defect density led to a higher carrier recombination rate [47]. Fig. 11(b) shows the effect of defect density at the ZnSnN_2_/Si interface on the PV parameters. In Fig. 11(b), it is evident that the output parameters of the solar cell remain nearly constant as the defect density increases from 10^7^ to 10^13^ cm^−2^. Consequently, the defects at interface II (Absorber/BSF) do not significantly impact the photovoltaic performance parameters [47].

### 3.6 Impact of parasitic resistances on photovoltaic performance

**Fig. 12:**
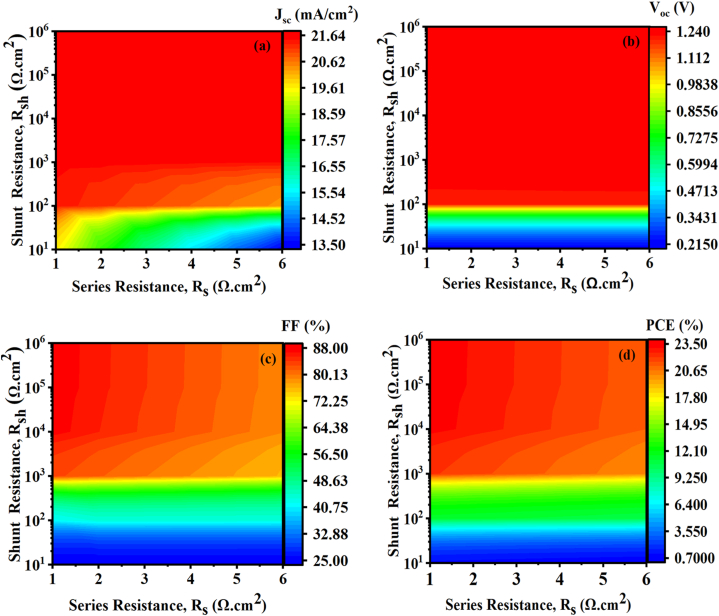
Contour plot of the effect of parasitic resistances (R_s_ and R_sh_) on (a) Jsc, (b) Voc, (c) FF, and (d) η.

Using SCAPS software, losses at the p-n junction could be considered. Additional research is required to explore potential applications of ZnSnN_2_ material. Our study focused on the impact of series and shunt resistances on the device. However, further investigation is necessary to fully demonstrate the advantages of this emerging ternary nitride semiconductor in the context of photovoltaic devices and optoelectronic applications. Fig. 12 shows the variation of R_s_ and R_sh_ of the new thin ZnSnN_2_ solar cell and the corresponding change in (a) Jsc, (b) Voc, (c) FF, and (d) η at ambient temperature. As discussed in Ref. [53], high shunt and low series resistance lead to high efficiency. Based on this condition, series, and shunt resistances have been studied to improve performance from 1 to 5 Ω cm^2^ and 10^1^–10^6^ Ω cm^2^, respectively. We can observe a similarity in the trends of J_SC_ (Fig. 12(a)), V_OC_ (Fig. 12(b)), FF (Fig. 12(c)), and PCE (Fig. 12(d)). In the case of J_SC_ (Fig. 12 (a)), the results indicate a maximum value of over 21.64 mA/cm^2^ when the series resistance (R_s_) is approximately 1 Ω cm^2^, and the shunt resistance (R_sh_) is greater than or equal to 10^2^ Ω cm^2^. Regarding V_OC_ (Fig. 12(b)), the results show a maximum value of over 1.24 V, regardless of the series resistance value, when the shunt resistance (R_sh_) is greater than or equal to 10^2^ Ω cm^2^. Similarly, FF (Fig. 12(c)) demonstrates a maximum value of over 88% when the shunt resistance (R_sh_) is greater than or equal to 10^3^ Ω cm^2^, irrespective of the series resistance value. Fig. 12(d) displays the efficiency (PCE), and it illustrates that the best efficiency of 23.9% is achieved when R_s_ = 1 Ω cm^2^ and R_sh_ = 10^6^ Ω cm^2^. Thus, achieving an ideal balance between series and shunt resistance is crucial for optimal solar cell performance.

### 3.7 Influence of back surface field (BSF) layer thickness on photovoltaic characteristics

**Fig. 13:**
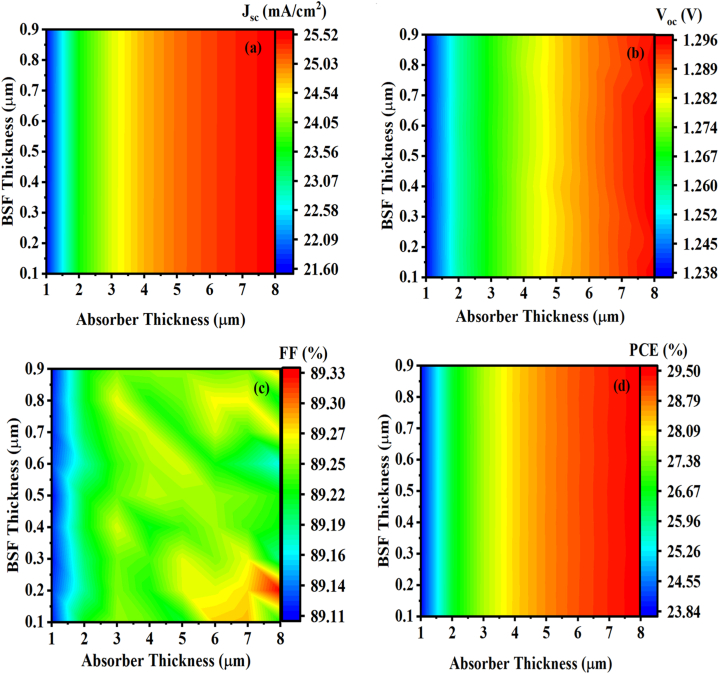
Contour plot of the effect of the thickness of the absorber layer and the thickness of the BSF layer on (a) Jsc, (b) Voc, (c) FF, and (d) η.

**Fig. 14:**
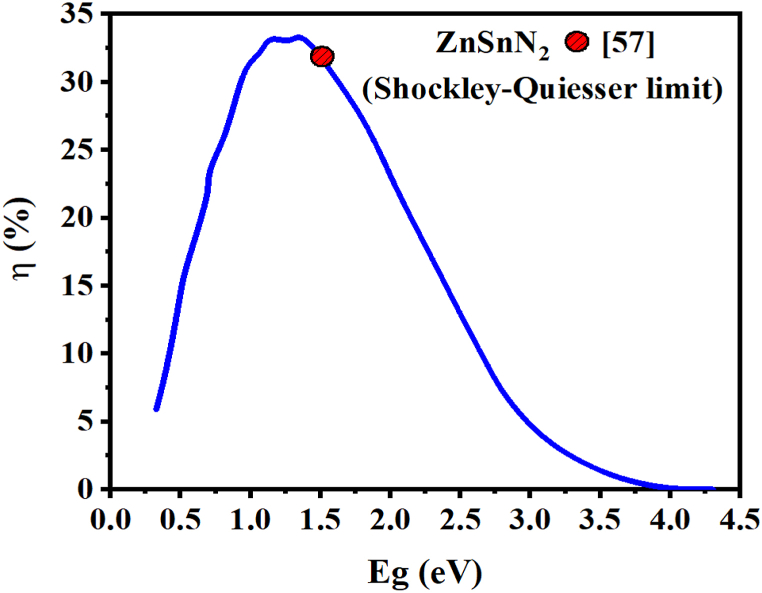
The detailed balance limit (or Shockley–Queisser limit) of power conversion efficiency as a function of band gap energy for single-junction solar cells [57].

**Fig. 15:**
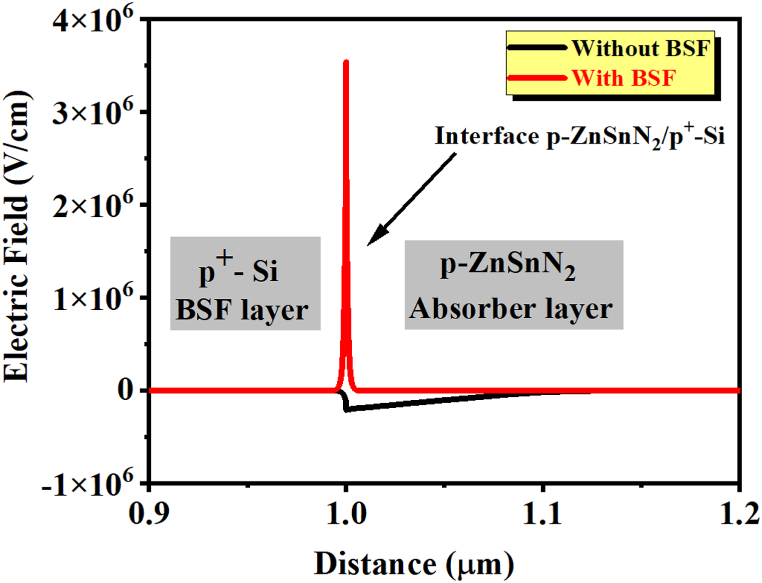
Electric field at the p + -Si/p-ZnSnN_2_ interface.

A back surface field layer with a higher doping concentration is added on the back side of a solar cell. This work combines ZnSnN_2_ with a thin silicon layer to act as BSF [27,54]. It has been demonstrated that incorporating a passivation layer may enhance the efficiency of collecting photogenerated carriers in photovoltaic devices [47]. As an element, silicon is highly abundant, beneficial, and cheaper than some III-V materials [55]. Under different absorbing thickness layers, the effect of variation in Si (BSF) layer thickness on cell performance is illustrated in Fig. 13. The thickness of the absorber layer varies between 1 μm and 8 μm, while the thickness of the Si layer varies between 0.1 μm and 0.9 μm. It can be observed that the variation in the thicknesses of the absorber layer and BSF layer affects the J_SC_ (Fig. 13(a)), V_OC_ (Fig. 13(b)), FF (Fig. 13(c)), and PCE (Fig. 13(d)) of the solar cell. It can be seen that the J_sc_ and η increase slightly with increasing Si thickness. In contrast, the absorber layer's thickness strongly affects the cell's performance [56]. Shockley-Queisser confirms the better performance of this ZnSnN_2_-based SC, as shown in Fig. 14. For the value of w_p_ (ZnSnN_2_) = 8 μm, we obtain an efficiency of η 29.5% (∼ 30%) as mentioned in Ref. [57]. A high absorption coefficient (α) of ∼10^5^ cm^−1^ made ZnSnN_2_ comparable to the III-V, I-II-IV-VI, and I-III-VI_2_ semiconductors [6,35,58,59]. During p-n junction formation, an electric field is formed at the interface ZnSnN_2_/Si on the rear face to lower surface recombination velocity, thereby improving the solar cells' electrical characteristics [60]. As a result of the 0.3 μm width of the back surface field layer and the 8 μm width of the absorber layer, the J_sc_ is 25.50 mA/cm^2^, with a conversion efficiency of 29.5%. On the other hand, in the case of a thin ZnSnN_2_ solar cell with a thickness of w_p_ = 1 μm, a P + -P junction is formed by incorporating a BSF layer (p^+^-Si) between the absorber layer (*p*-ZnSnN_2_) and the rear side of the cell. This configuration results in a significant electric field strength of approximately 3.56 MV/cm, as determined through a SCAPS software analysis [61,62]. This information is visually represented in Fig. 15. The high electric field in the p + -p junction effectively reflects minority electrons from the back surface. This reflection mechanism helps increase the J_sc_ by reducing the dark current, as discussed in Ref. [63].

### 3.8 Optimized ultrathin ZnSnN_2_ solar cells

**Fig. 16:**
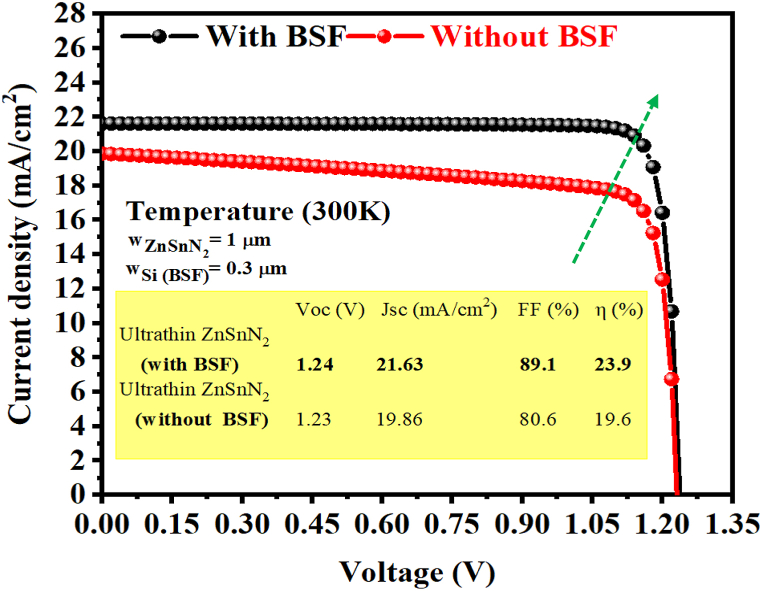
J-V curves of the new thin ZnSnN_2_ SC with and without BSF.

Table .6 displays the photovoltaic parameters that we have obtained based on ultrathin ZnSnN_2_ structure solar cells compared with CIGS [64] and CZTS [65] ultrathin structures solar cells, where w_p_ ∼ 500 nm. In Table .5, the ZnSnN_2_ ultrathin solar cell shows the best performance over CIGS and CZTS, with an efficiency of 20.08%. Fig. 1(a) depicts the thin ZnSnN_2_ solar cell's structure without BSF. Fig. 16 shows the J-V characteristics of the thin ZnSnN_2_ SC without BSF and with a BSF layer in illumination and w_p_ (ZnSnN_2_) = 1 μm. Using the parameters listed in Table 1, Table 2, Table 3, we obtained the J-V characteristic at room temperature. After inserting BSF, improvement in J_sc_ 19.86–21.63 mA/cm^2^ and efficiency 19.6% to 23.9% (about Δη = 4.3%) was observed (Table .7). Therefore, inserting the BSF layer reduces recombination at the rear surface, enhancing carrier collection through efficient charge carrier extraction and reducing surface recombination velocity by increasing V_oc_ and FF [66,67]. The optimal results were achieved with J_sc_ = 21.63 mA/cm^2^, V_oc_ = 1.24 V, FF = 89.1%, and PCE ∼24% with an optimal BSF layer thickness of 0.3 μm.

**Table 6:** PV parameters for ultrathin ZnSnN_2_ structure solar cells compared with CIGS and CZTS ultrathin solar cells (w_p_∼500 nm).

Ultrathin structure (w_p_ ∼500 nm)	Voc (V)	Jsc (mA/cm^2^)	FF (%)	η (%)
Ultrathin ZnSnN_2_ (This work)	1.215	18.56	88.95	20.08
Ultrathin CIGS [64]	0.771	28.5	81.7	18.00
Ultrathin CZTS [65]	0.701	19.8	67	9.3

**Table 7:** Comparison between performances of thin solar cells with and without Si BSF layer, where w_p_ (w_ZnSnN2_) = 1 μm.

Thin structure (w_p_ = 1 μm)	Voc (V)	Jsc (mA/cm^2^)	FF (%)	η (%)
Thin ZnSnN_2_ (with BSF)	1.24	21.63	89.1	∼24
Thin ZnSnN_2_ (without BSF)	1.23	19.86	80.64	19.6

### 3.9 Generation and recombination mechanism

**Fig. 17:**
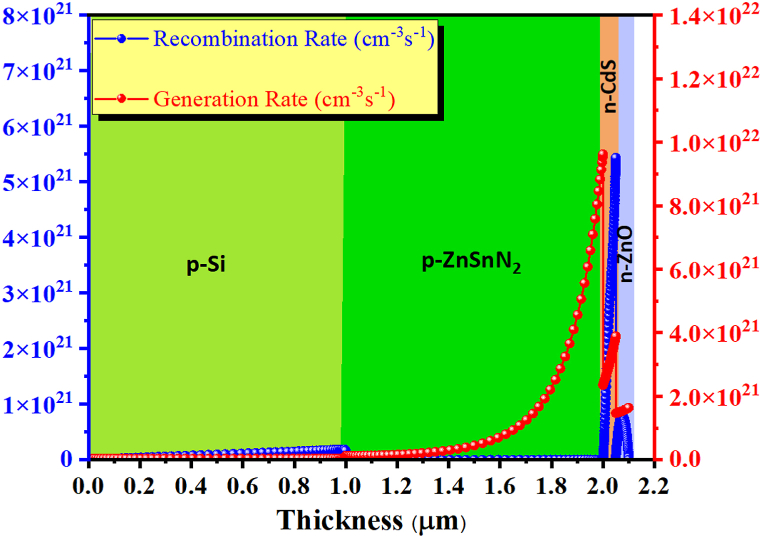
Variation of carrier recombination and generation rates of the thin ZnSnN_2_ solar cell structure.

This section analyzes the generation and recombination mechanisms of the thin ZnSnN_2_ structure. It can be seen in Fig. 17 that the highest generation rate is achieved for the proposed thin solar cell when w_p_ = 1 μm, w_BSF_ = 1 μm, and N_t_ (ZnSnN_2_) = 10^10^ cm^−3^. The maximum generation rate (9.62 × 10^21^cm^−3^s^−1^) was found at positions of ∼2 μm. This is because the absorption rate of photons at this particular position in the studied cell is higher than that of other positions. Consequently, the maximum generation rate describes the maximum number of electrons generated at a specific location. On the other hand, recombination is the inverse process of generation, during which electrons and holes can recombine and mutually annihilate [68]. Depending on the type of recombination, this can involve carriers from the conduction band, the valence band, or intermediate energy levels [66]. As previously stated, defects in each layer of the proposed cell also affect electron-hole recombination. The maximum recombination rate for the cell (5.46 × 10^21^cm^−3^s^−1^) was also observed at positions of 2 μm, similar to the generation rate.

### 3.10 Impact of the front contact and back contact work function on photovoltaic parameters

**Fig. 18:**
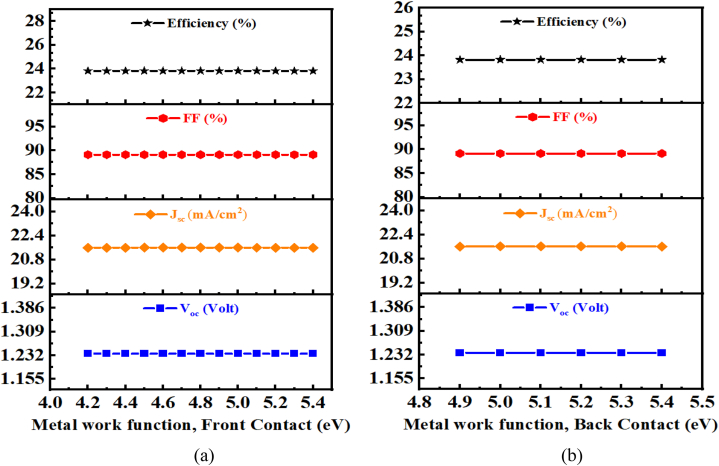
Effects of (a) front contact metal work function and (b) back contact work function on PV performance.

This section analyzes the effect of the front contact and back contact work function on the performance of thin ZnSnN_2_ SCs. With an optimal BSF layer thickness of 0.3 μm, w_p_ = 1 μm, and N_t_ (ZnSnN_2_) = 10^10^ cm^−3^, Fig. 18 (a,b) illustrates the impact of different front and back metal work functions. The results indicate that increasing the front metal work function from 4.2 to 5.4 eV and the back metal work function from 4.9 to 5.4 eV does not significantly affect cell performance [69,70]. Optimizing the metal work functions and other parameters to achieve the highest performance for a specific solar cell design often requires a combination of experimental characterization and modeling [69]. In Ref. [27], Molybdenum is mentioned as the back contact material, and this study also employs Molybdenum as the back contact. According to the simulation results, using either the flat-band approximation or the real front-contact metal and back-contact work function values yields the same results. However, the impact of the front metal work function may be negligible due to other factors affecting the cell performance [71].

### 3.11 An overview of theoretical and experimental research on ZnSnN_2_ solar cells

The structures based on ZnSnN_2_ semiconductor and their corresponding conversion efficiencies are listed in Table .8 [[15], [16], [17], [18],^72^,^73^]. Recent theoretical and experimental studies on various ZnSnN_2_ solar cells demonstrate the suitability of this material for enhancing efficiency and reducing costs, as shown in Table 8. It has been recognized that developing alternative materials, such as ZnSnN_2_, is of utmost importance and poses a significant challenge. Moreover, the potential applications of ZnSnN_2_ extend beyond solar cells and encompass various optoelectronic devices. For instance, it can be employed as a light emitter, offering a stable functionality due to its low mismatch (or low strain) with wurtzite III-N materials, demonstrating exceptional compatibility [4,[74], [75], [76], [77], [78]]. The nomenclature used throughout this paper is listed in Table .9.

**Table 8:** Functional characteristics of experimental and simulated ZnSnN_2_ Solar cells.

ZnSnN_2_ structure	Year	PCE (%)	Reference
CuCrO_2_/ZnSnN_2_ (Simulation)	2018 2020	23 22	[16] [17]
SnO/ZnSnN_2_ (Experimental) SnO/Al_2_O_3_/ZnSnN_2_ (Experimental)	2018 2018	0.37 1.54	[72] [15]
Cu_2_O/ZnSnN_2_ (Experimental)	2023	0.18	[73]
CdS/ZnSnN_2_ (Simulation) CdS/ZnSnN_2_/Si (Simulation)	2021 2023	22 23.9–29.5	[18] This work

**Table 9:** Nomenclature table with units.

Nomenclature		Units
ZnSnN_2_	Zinc tin nitride	
Si	Silicon	
CIGS	Copper indium gallium diselenide	
CZTS	Copper zinc tin sulfide	
ZnO	Zinc oxide	
CdS	Cadmium sulfide	
Mo	Molybdenum	
BSF	Back surface field	
SCs	Solar cells	
HJT	Heterojunction solar cell	
FDM	Finite difference method	
SRV	Surface recombination velocity	cm/s
QE	Quantum efficiency	%
CBO	Conduction band offset	eV
VBO	Valence band offset	eV
T	Temperature	K
R_s_	Series resistance	Ω.cm^2^
R_sh_	Shunt resistance	Ω.cm^2^
N_t_	Defect density	cm^−3^
V_bi_	Built-in voltage	V
V_oc_	Open-circuit voltage	V
J_sc_	Short-circuit current	mA/cm^2^
FF	Fill factor	%
PCE (η)	Power conversion efficiency	%

## 4 Conclusion

This study employed SCAPS-1D software to investigate the electrical properties of ZnSnN_2_ solar cells. Numerous factors were analyzed, encompassing the thickness of the window layer, buffer layer, absorber layer, and BSF layer, as well as the operating temperature, parasitic resistances (series and shunt), defect density at the interfaces, and the absorber layer, electric field, contacts work function, and generation-recombination profile. The parameters were optimized under room temperature, one sun AM1.5G, and flat-band conditions on the front contact. The study's results demonstrated that a thin solar cell with a thickness of 1 μm achieved an efficiency of 23.9% under optimal conditions. For a practical solar cell with a thicker absorber at room temperature, the following optimal values were determined: w_p_ = 8 μm, w_BSF_ = 0.3 μm, R_sh_ = 10^6^ Ω cm^2^, R_s_ = 1 Ω cm^2^, and a low defect density in the ZnSnN_2_ semiconductor (N_t_ = 10^10^ cm^−3^), resulting in a significantly higher efficiency of 29.5%. Compared to conventional thin-film solar cells like CIGS and CZTS, ZnSnN_2_-based structures offer distinct advantages, including a high absorption coefficient (10^5^ cm^−1^) and high efficiency (∼30%). Consequently, developing environmentally friendly and cost-effective materials such as ZnSnN_2_ is paramount and poses a significant challenge for future advancements in solar technology. However, additional investigations are required to address limitations and explore further factors. This study offers valuable insights into the p-type characteristics of ZnSnN_2_, illuminating an area of research that requires further attention and development. Furthermore, this study emphasizes the significant potential of this material for various optoelectronic devices. Finally, as a promising perspective, we propose the incorporation of ZnSnN_2_ in tandem solar cells, in conjunction with Si, to attain high efficiency. This proposition is based on recent developments in the field.

## Author contribution statement

Abdelmoumene Laidouci: Mamta Mamta: Vidya Nand Singh: Conceived and designed the analysis; Analyzed and interpreted the data; Contributed analysis tools or data; Wrote the paper.

Pratap Kumar Dakua, Deepak Kumar Panda: Analyzed and interpreted the data; Wrote the paper.

## Funding statement

This research received no specific grant from public, commercial, or not-for-profit funding agencies.

## Data availability statement

Data included in article/supplementary material/referenced in the article.

## Declaration of competing interest

The authors declare that they have no known competing financial interests or personal relationships that could have appeared to influence the work reported in this paper.
